# Comparative and Phylogenetic Analyses of Ginger (*Zingiber officinale*) in the Family Zingiberaceae Based on the Complete Chloroplast Genome

**DOI:** 10.3390/plants8080283

**Published:** 2019-08-12

**Authors:** Yingxian Cui, Liping Nie, Wei Sun, Zhichao Xu, Yu Wang, Jing Yu, Jingyuan Song, Hui Yao

**Affiliations:** 1 Key Lab of Chinese Medicine Resources Conservation, State Administration of Traditional Chinese Medicine of the People’s Republic of China, Institute of Medicinal Plant Development, Chinese Academy of Medical Sciences and Peking Union Medical College, Beijing 100193, China; 2 Institute of Chinese Materia Medica, China Academy of Chinese Medical Sciences, Beijing 100700, China; 3 Engineering Research Center of Chinese Medicine Resources, Ministry of Education, Beijing 100193, China

**Keywords:** *Zingiber officinale*, Zingiberaceae, complete chloroplast genome, comparative analysis, phylogenetic analysis

## Abstract

*Zingiber officinale*, commonly known as ginger, is an important plant of the family Zingiberaceae and is widely used as an herbal medicine and condiment. The lack of chloroplast genomic information hinders molecular research and phylogenetic analysis on ginger. We introduced the complete chloroplast genome of *Z. officinale* and identified its phylogenetic position in Zingiberaceae. The chloroplast genome of *Z. officinale* is 162,621 bp with a four-part circular structure and 36.1% GC content. All 113 unique genes were annotated. A total of 78 simple sequence repeats (SSRs) and 42 long repeat sequences, which are potential areas for species authentication, were found. Comparative analysis revealed some highly variable regions, including *rps16-trnQ-UUG*, *atpH-atpI*, *trnT-UGU-trnL-UAA*, *ycf1*, and *psaC-ndhE*. Moreover, the small single-copy (SSC) region was the most variable region in all four shared regions, indicating that it may be undergoing rapid nucleotide substitution in the family Zingiberaceae. Phylogenetic analysis based on all available chloroplasts of Zingiberales in the National Center for Biotechnology Information indicated that *Zingiber* is a sister branch to *Kaempferia* species. The availability of the *Z. officinale* chloroplast genome provided invaluable data for species-level authentication and phylogenetic analysis and can thus benefit further investigations on species in the family Zingiberaceae.

## 1. Introduction

*Zingiber officinale*, commonly known as ginger, is a common plant of the family Zingiberaceae that is universally cultivated in the Central, Southeastern, and Southwestern provinces of China, as well as in tropical Asia [1]. Its rhizome is a common plant material with medicinal and condiment properties. In China, its fresh and dried forms with different therapeutic effects are recorded in the Chinese Pharmacopoeia [2]. Currently, ginger has gained attention as researchers has demonstrated that its compounds can be used to treat cardiovascular disease [3,4] and have antioxidant [5,6], anti-inflammatory [4,7], analgesic, immunomodulatory, antitumorigenic [7,8], and anti-emetic [9] properties with few side effects. Increasing attention has focused on the genetic engineering and molecular research of ginger in recent years as a result of its medicinal effects and edibility [10]. However, to the best of our knowledge, the chloroplast genome of ginger is still unclear. Only the chloroplast genome sequences of six species in the whole family Zingiberaceae were recorded in the National Center for Biotechnology Information (NCBI). Hence, more information should be obtained about the chloroplast genome resources of the Zingiberaceae species.

The complete chloroplast genome of angiosperms usually consists of four parts: A large single-copy (LSC) region, small single-copy (SSC) region, and two similar inverted repeat (IR) regions [11]. Unlike the nuclear genome, the chloroplast genome of most angiosperms has the characteristics of maternal inheritance, highly conserved gene content and order, and lower recombination rate, making it an ideal material for species authentication and phylogenetic studies [12,13,14,15]. Highly variable regions on the chloroplast genomes have been revealed and successfully used for species authentication, such as in *Rhubarb botanical* [16] and *Ipomoea* species [17]. Moreover, some studies have suggested that complete chloroplast genomes can be used as the super-code to identify related species, which is a challenge when using traditional molecular markers, such as in the case of *Ephedra* species [18], grass species [19], and *Dendrobium* species [20]. The availability of complete chloroplast genomes has greatly contributed to resolving the evolutionary and phylogenetic relationships of angiosperms [19,21,22].

Here, we introduce the complete chloroplast genomes of *Z. officinale* and compare the obtained sequences with reported chloroplast genomes in the family Zingiberaceae. The availability of the *Z. officinale* chloroplast genome will greatly contribute to species authentication, phylogenetic analysis, and genetic engineering study of species in the family Zingiberaceae.

## 2. Results and Discussion

### 2.1. Zingiber Officinale Chloroplast Genome Features

**Figure 1:**
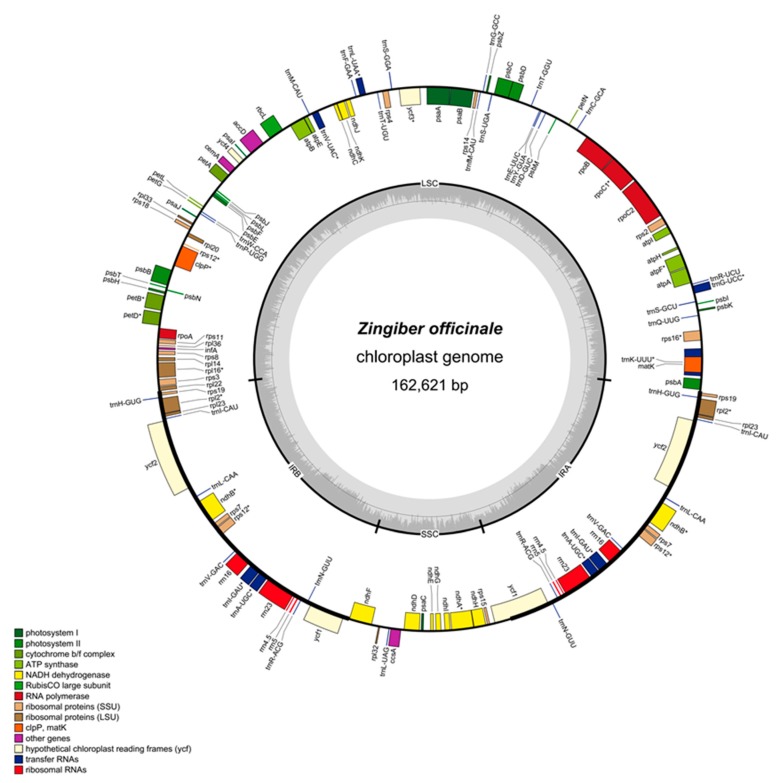
Gene map of the *Zingiber officinale* complete chloroplast genome. Genes that are inside and outside the circle are transcribed clockwise and counterclockwise, respectively. The darker gray area in the inner circle corresponds to GC content, whereas the lighter gray area corresponds to AT content.

Approximately 8.5 Gb of raw data from *Z. officinale* with pair-end 150 bp read lengths were obtained. The *Z. officinale* chloroplast genome is 162,621 bp and contains four regions, including an LSC (87,486 bp) region, SSC (15,577 bp) region, and two identical IR (29,779 bp) regions, as shown in Table 1 and Figure 1. The overall GC contents of the *Z. officinale* chloroplast genome and protein-coding regions were 36.1% and 37.1%, respectively. Both IR (41.1%) regions possess all four rRNA genes with high GC content in the genome–higher than those of the LSC (33.8%) and SSC (29.7%) regions (Table 1). The AT content (71.1%) in the third codon position was remarkably higher than those in the first (55.2%) and second codon positions (62.2%) within the protein-coding regions of *Z. officinale* chloroplast genomes (Table 1). These observations were similar to those for other reported plants [21,23,24,25] and were used as identification characteristics to discern chloroplast DNA from nuclear and mitochondrial DNA. 

Table 2 shows the 113 unique genes that were identified in the *Z. officinale* chloroplast genome, which included 79 protein-coding genes, 30 tRNA genes, and four rRNA genes. Twenty genes (eight protein-coding genes, eight tRNA genes and four rRNA genes) were duplicated in the IR regions, resulting in 133 genes located in the complete *Z. officinale* chloroplast genome. The LSC regions contained 60 protein-coding genes and 21 tRNA genes, whereas the SSC regions contained 11 protein-coding genes and one tRNA gene. A total of 10 protein-coding genes and seven tRNA genes contained one intron, whereas three genes (*rps12*, *trnK-UUU*, *ycf3*) contained two introns, as shown in Table S1. Among them, the *trnK-UUU* gene has the largest intron, with a size of >2500 bp, as it contains the *matK* gene. 

### 2.2. Codon Usage Analysis

**Figure 2:**
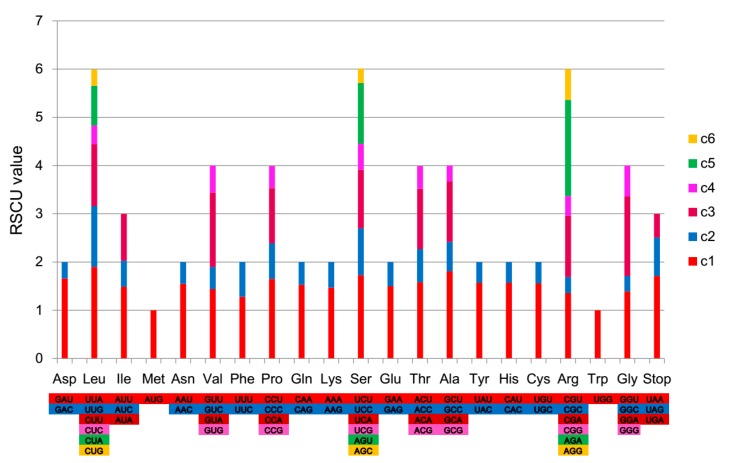
Codon content of all protein-coding genes in the *Z. officinale* chloroplast genome.

The chloroplast genome of *Z. officinale* was analyzed for its codon usage frequency based on the sequence of protein-coding genes and on relative synonymous codon usage (RSCU). RSCU refers to the relative probability of a codon encoding a corresponding amino acid synonymous codon, which removes the effect of amino acid composition on codon usage. The RSCU value of this codon is equal to one if codon use is not preferred, whereas a value greater than one means a relatively large number of codons, and vice versa. A total of 63 codons encoded 20 amino acids. All annotated genes of *Z. officinale* were encoded by 26,389 codons, as shown in Figure 2 and Table S2. Similarly shown in this figure and table is that among the amino acids, leucine, with 2732 (10.4%) codons, occurred most frequently, whereas cysteine 303 (1.1%) was observed to have the least occurrence. Most of the amino acid codons have preferences except for methionine (AUG) and tryptophan (UGG), whose RSCU values are equal to one. A majority of the preferred synonymous codons (RSCU > 1) possess A- or U-ending codons, except for *trnL-CAA*, which is encoded by UUG (Figure 2 and Table S2). Codons ending with A and/or U accounted for 71.2%, resulting in the bias for A/T bases. Similar patterns were observed in other reported chloroplast genomes, such as in *Ulmus* [26], *Papaver* [27], *Lycium* [28], and *Taxillus* [11] species.

### 2.3. Repeat Structure Analysis

Simple sequence repeats (SSRs), also called microsatellites, are present throughout genomes and consist of tandem repeats of one to six nucleotides [29]. Most SSRs have high levels of polymorphism and are widely used for species authentication, phylogenetic analysis, and population genetics [17,30,31,32]. In total, 78 SSRs were identified in the *Z. officinale* chloroplast genome, including 27 mononucleotide, 23 dinucleotide, five trinucleotide, 21 tetranucleotide, and two pentanucleotide repeats (Table 3 and Table S3). Among them, 54, 14, and five SSRs were distributed in the LSC, SSC, and IR regions, respectively. A/T mononucleotide repeats (34.6%) were observed to be most common, followed by AT/TA dinucleotide repeats (26.9%), as shown in Table 3. Furthermore, A and T were the most frequent bases in all SSR types, which resulted in the bias in base in the *Z. officinale* chloroplast genome.

**Figure 3:**
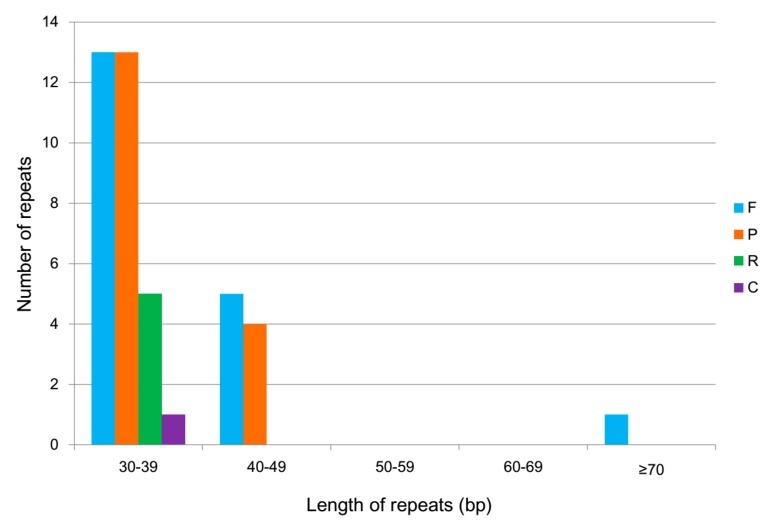
Repeat sequences in the *Z. officinale* chloroplast genome. F, forward; P, palindrome; R, reverse; and C, complement.

Some >30 bp structures are known as long repeat sequences, which might be helpful to phylogenetic analysis and which increase chloroplast genome rearrangement [33]. Figure 3 shows 42 long repeats in the *Z. officinale* chloroplast genome. Of the identified repeats, 32 have sizes between 30 and 39 bp, including 13 forward, 13 palindromic, five reverse, and one complement repeat. Moreover, five forward and four palindromic repeats are 40–49 bp, whereas one forward repeat is more than 70 bp.

### 2.4. Comparative Analysis

**Figure 4:**
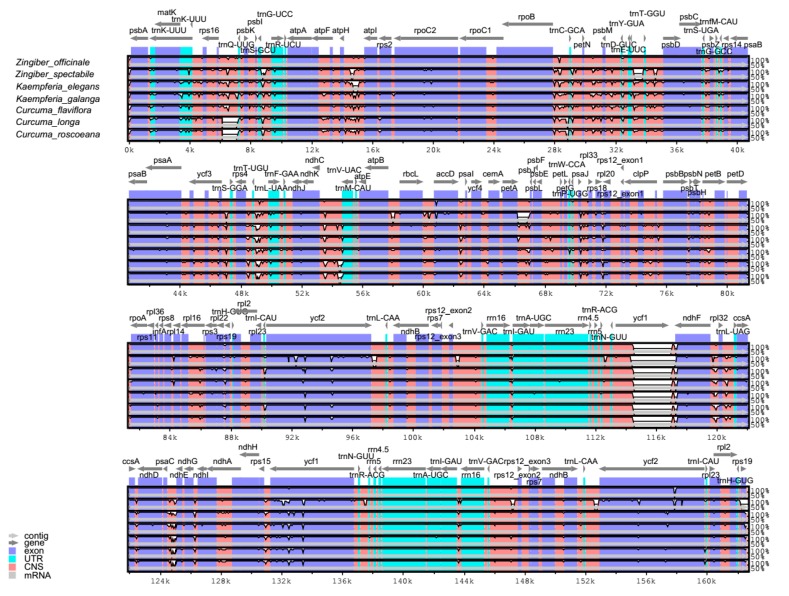
Comparison of the complete chloroplast genomes of seven species in the family Zingiberaceae using mVISTA, with *Z. officinale* as a reference. Blue block, conserved genes; sky-blue block, transfer RNA (tRNA) and ribosomal RNA (rRNA); and red block, conserved noncoding sequences (CNS). White represents regions with sequence variation among the seven species.

The *Z. officinale* chloroplast genome was compared to those of species in the family Zingiberaceae (*Z. spectabile*, *Kaempferia elegans*, *K. galangal*, *Curcuma longa*, *C. flaviflora*, *C. roscoeana*), with the annotated *Z. officinale* sequence as a reference (Figure 4). Among these species, *Z. officinale* had the third longest chloroplast genome, following *K. elegans* and *K. galangal*. It is larger than that of *Z. spectabile* by 6731 bp. The result showed that the non-coding regions appeared to be more variable than the coding regions, the two IR regions were less divergent than that LSC and SSC regions, four rRNA genes were the most conserved regions, and the intergenic spacers were the most divergent regions. The highly divergent regions among the seven chloroplast genomes occurred in the intergenic spacers, including *rps16-trnQ-UUG*, *atpH-atpI*, *trnT-UGU-trnL-UAA*, *ycf1*, and *psaC-ndhE*, regions of which had been also observed in other plant chloroplast genomes in Zingiberaceae [21,24,28]. Moreover, *ycf1*, *ycf2*, and *ndhF* were the most variable coding regions in these species.

**Figure 5:**
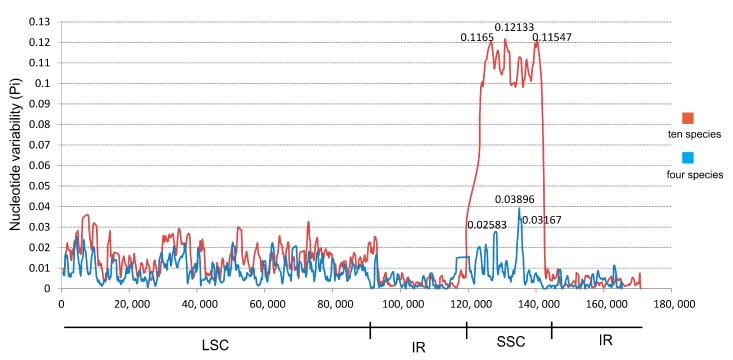
Sliding window analysis of the *Z. officinale* chloroplast genome. The red line indicates the comparison of 10 species in the family Zingiberaceae (*Z. officinale*, *Z. spectabile*, *K. elegans*, *K. galangal*, *C. longa*, *C. flaviflora*, *C. roscoeana*, *Amomum compactum*, *A. krervanh*, and *Alpinia oxyphylla*); the blue line indicates the comparison of four species in the family Zingiberaceae (*Z. officinale*, *Z. spectabile*, *K. elegans*, and *K. galang*). Window length: 800 bp; step size 200 bp. *x* axis: Position of the midpoint of a window. *y* axis: nucleotide diversity of each window.

Moreover, 10 species in Zingiberaceae (*Z. officinale*, *Z. spectabile*, *K. elegans*, *K. galangal*, *C. longa*, *C. flaviflora*, *C. roscoeana*, *Amomum compactum*, *A. krervanh*, and *Alpinia oxyphylla*) were observed to have highly variable regions in their chloroplast genomes by sliding window analysis using DnaSP software (Figure 5). The results showed that the average value of nucleotide variability (PI) of all the 10 species was 0.0187, and that of the four relatively related species (*Z. officinale*, *Z. spectabile*, *K. elegans*, and *K. galang*) was 0.0075. The IR regions were observed to have lower PI value than the other regions. Moreover, the SSC regions were the most variable among all four regions of the complete chloroplast genomes in Zingiberaceae. Furthermore, some regions with high PI values (>0.115 or >0.025) were observed in the SSC region. The results indicated that the SSC region may be undergoing rapid nucleotide substitution in species of family Zingiberaceae and may contain more important variable information for species authentication and phylogenetic analysis.

### 2.5. Phylogenetic Analysis

**Figure 6:**
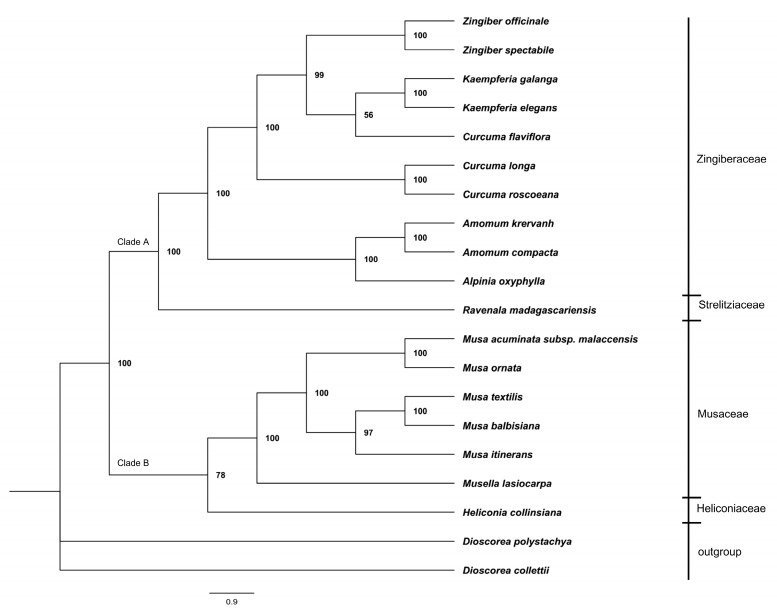
Likelihood (ML) phylogenetic tree of 19 species in order Zingiberales based on the concatenated sequences of 75 shared protein-coding genes of chloroplast genomes. Numbers at nodes are values for bootstrap support. *Dioscorea polystachya* and *D. collettii* were used as outgroups.

In recent years, more chloroplast genome sequences have been identified using rapid and high-throughput sequencing [34,35]. The continuously expanding chloroplast genome database provides an important basis for the determination of the evolutionary and phylogenetic relationships of plants. Here, to reveal the phylogenetic position of *Z. officinale* in Zingiberales, 19 plasmid genomes were downloaded from GenBank, which contained all the available chloroplast genomes in Zingiberales. From phylogenetic analysis (Figure 6), Zingiberales species were basically divided into two branches (bootstrap value = 100%): Clade A and Clade B. On the one hand, Clade A consisted of species in the Zingiberaceae and Strelitziaceae families, indicating that these families had a closer relationship in Zingiberales. *Z. officinale* is a sister species to *Z. spectabile* with bootstrap values of 100% from the same clade. The result also provided evidence that *Zingiber* species are a sister-branch to the *Kaempferia* species. Species in *Zingiber*, *Kaempferia*, and *Curcuma* being clustered into a phylogenetic group demonstrated a close relationship among them. Two species in *Amomum* were clustered into a branch with *Alpinia oxyphylla*. On the other hand, Clade B contained the Musaceae and Heliconiaceae families and all species in *Musa* clustered into a monophyletic clade, which had a close relationship with *Musella lasiocarpa*, followed by *Heliconia collinsiana*. The results also indicated that the chloroplast genome database is helpful to phylogenetic analysis of species in the family Zingiberaceae. It will also be a useful resource for molecular phylogeny studies within the order Zingiberales.

## 3. Materials and Methods

### 3.1. Plant and DNA Sources

Fresh *Z. officinale* leaves were collected from Laiwu City, Shandong Province, China, and were identified by Prof. Yulin Lin from the Institute of Medicinal Plant Development (IMPLAD), Chinese Academy of Medical Sciences (CAMS). The voucher specimens were deposited in the herbarium of IMPLAD. Total genomic DNA was extracted from the clean sample leaves using a DNeasy Plant Mini Kit following standard protocol (Qiagen Co., Hilden, Germany) and the DNA concentration and quality were respectively assessed through Nanodrop 2000C spectrophotometry and electrophoresis in 1% (*w/v*) agarose gel. Approximately 1 μg genomic DNA was used for sequencing library construction. Paired-end libraries with insert sizes of 400 bp were prepared following Illumina’s standard genomic DNA library preparation procedure. Total DNA was sequenced in Illumina HiSeq X. 

### 3.2. Chloroplast Genome Assembly and Annotation

Raw sequencing data were generated by Illumina and the low-quality reads were trimmed using Trimmomatic v0.36 software [36] with default parameters. Clean data obtained by the above quality control processes were used to assemble the chloroplast genome. All available chloroplast genomes of plants recorded in NCBI were downloaded to construct a basic local alignment search tool (BLASTn) database for mapping. Then, BLASTn with the default parameter was used to map and compare obtained reads to the reference chloroplast database and screen the chloroplast-like reads. Next, these reads were assembled using ABySS 2.0 [37] with multiple-Kmer parameters, and the optimal results of the assembly were obtained. SOAPdenovo2 [38] and SSPACE [39] were used to create the contigs and scaffolds of the chloroplast genome, respectively. GapCloser [38,40] software was subsequently applied to fill up the remaining local inner gaps and correct the single base polymorphism for the final assembly results. To check the accuracy of the assembly, four junctions between the IRs and SSC/LSC regions were verified by PCR amplification and Sanger sequencing using newly designed primers (Table S4). Then, the complete chloroplast genome was acquired.

CPGAVAS [41] and DOGMA [42] were used to annotate the complete chloroplast genome with default settings followed by manual corrections. tRNAscan-SE [43] software was applied to identify the tRNA genes and BLAST search was used to annotate boundaries of genes, introns/exons and coding regions. The online software OGDRAW v1.2 [44] was programed to draw the circular map of the complete chloroplast. Finally, the complete and correct chloroplast genome of *Z. officinale* was submitted to GenBank with the accession number MH161428.

### 3.3. Chloroplast Genome Structure and Comparison Analysis

The software MEGA6.0 [45] was used to calculate the GC content and investigate the distribution of codon usage with the RSCU ratio. The software MISA [46] was used to detect SSRs with the parameters set to 10 repeat units (>10) for mononucleotide SSRs, five repeat units (>5) for dinucleotide, four repeat units (>4) for trinucleotide, and three repeat units (>3) for tetranucleotide, pentanucleotide, and hexanucleotide SSRs. The size and location of repeat sequences, including forward, palindromic, reverse, and complement repeats, were identified by REPuter [47]. For all repeat types, the minimal size was 30 bp and the two repeat copies had at least 90% similarity. The whole chloroplast genomes were aligned using the online software MAFFT [48]. Whole-genome alignment of *Z. officinale* and other species in Zingiberaceae downloaded from Genbank was compared by mVISTA [49] and calculated suing DnaSP [50] software to determine the nucleotide diversity with 200 bp step size and 800 bp window length. 

### 3.4. Phylogenetic Analyses

For phylogenetic analysis, a total of 19 complete chloroplast genomes were downloaded from NCBI (Table S5), and 75 common protein-coding genes shared in these chloroplast genomes were applied to construct the ML phylogenetic tree. Firstly, all shared genes from these chloroplast genomes were extracted and aligned separately by multiple alignment using MAFFT [48] and were manually adjusted. Secondly, all aligned gene sequences were concatenated and verified. Lastly, phylogenetic trees were reconstructed based on 75 concatenated protein-coding gene sequences by ML methods with a bootstrap of 1000 repetitions. IQ-TREE [51] software was employed to find the best substitution model and construct the ML phylogenetic tree.

## 4. Conclusions

Here, we sequenced and analyzed the *Z. officinale* chloroplast genome. Firstly, the basic structures, gene information, and codon usage pattern were revealed. Secondly, 78 SSRs and 42 long repeat sequences were identified. Thirdly, a comparative analysis within the family Zingiberaceae was executed and some variable regions which have the potential to become DNA markers were revealed. The results showed that SSC was the most variable region and may be undergoing rapid nucleotide substitution in Zingiberacelae species. The ML tree indicated that the *Zingiber* species has a close relationship with species in *Kaempferia* and *Curcuma* and clearly show a phylogenetic relationship with species in the family Zingiberaceae, or even the order Zingiberacelae, through molecular methods. This study provided invaluable data for species authentication and phylogenetic analysis of plants in the family Zingiberaceae.

## Figures and Tables

**Table 1:** Base composition of the *Z. officinale* chloroplast genome.

Region	Positions	T(U) (%)	C (%)	A (%)	G (%)	Length (bp)
Total		32.2	18.4	31.7	17.7	162,621
IRa		30.1	21.3	28.8	19.8	29,779
IRb		28.8	19.8	30.1	21.3	29,779
LSC		33.7	17.4	32.4	16.5	87,486
SSC		34.3	15.8	36.0	13.9	15,577
CDS		31.7	17.2	31.2	19.9	79,167
	1st position	24	18.3	31.3	26.5	26,389
	2nd position	33	20.1	29.7	17.7	26,389
	3rd position	39	13.2	32.6	15.6	26,389

**Table 2:** Gene content of the *Z. officinale* chloroplast genome.

Group of Genes	Gene Names	Amount
Photosystem I	*psaA*, *psaB*, *psaC*, *psaI*, *psaJ*	5
Photosystem II	*psbA*, *psbB*, *psbC*, *psbD*, *psbE*, *psbF*, *psbH*, *psbI*, *psbJ*, *psbK*, *psbL*, *psbM*, *psbN*, *psbT*, *psbZ*	15
Cytochrome b/f complex	*petA*, petB*, petD*, *petG*, *petL*, *petN*	6
ATP synthase	*atpA*, *atpB*, *atpE*, atpF*, *atpH*, *atpI*	6
NADH dehydrogenase	ndhA*, ndhB*(×2), *ndhC*, *ndhD*, *ndhE*, *ndhF*, *ndhG*, *ndhH*, *ndhI*, *ndhJ*, *ndhK*	12
RubisCO large subunit	*rbcL*	1
RNA polymerase	*rpoA*, *rpoB*, rpoC1*, *rpoC2*	4
Ribosomal proteins (SSU)	*rps2*, *rps3*, *rps4*, *rps7*(×2), *rps8*, *rps11*, rps12** (×2), *rps14*, *rps15*, rps16*, *rps18*, *rps19*(×2)	15
Ribosomal proteins (LSU)	rpl2*(×2), *rpl14*, rpl16*, *rpl20*, *rpl22*, *rpl23*(×2), *rpl32*, *rpl33*, *rpl36*	11
Proteins of unknown function	*ycf1*(×2), *ycf2*(×2), ycf3**, *ycf4*	6
Transfer RNAs	38 *tRNA*s (8 in the IRs(×2)) **	38
Ribosomal RNAs	*rrn4.5*(×2), *rrn5*(×2), *rrn16*(×2), *rrn23*(×2)	8
Other genes	*accD*, clpP*, *matK*, *ccsA*, *cemA*, *infA*	6

(×2) indicates that the number of the repeating unit is 2; * indicates introns of genes.

**Table 3:** Types and amounts of simple sequence repeats (SSRs) in the *Z. officinale* chloroplast genome.

SSR Type	Repeat Unit	Amount	Ratio (%)
Mono	A/T	27	100.0
Di	AG/CT	2	8.7
	AT/TA	21	91.3
Tri	AAG/CTT	1	20.0
	AAT/ATT	4	80.0
Tetra	AAAC/GTTT	1	4.8
	AAAG/CTTT	4	19.0
	AAAT/ATTT	13	61.9
	AACT/AGTT	1	4.8
	AATG/ATTC	2	9.5
Penta	AAAAT/ATTTT	1	50.0
	AATAG/ATTCT	1	50.0

## Supplementary Materials

The following are available online at https://www.mdpi.com/2223-7747/8/8/283/s1, Table S1: Gene with introns in *Z. officinale* chloroplast genome, Table S2: Codon usage of *Z. officinale* chloroplast genome, Table S3: Simple sequence repeats (SSRs) in *Z. officinale* chloroplast genome, Table S4: Primer sequences at the boundaries between single copy and IR regions of *Z. officinale*, Table S5: GenBank accession numbers of 19 complete chloroplast genome sequences used for ML phylogenetic analyses.

## Abbreviations

LSC	large single copy
SSC	small single copy
IR	inverted repeat
ML	maximum likelihood
SSR	Simple sequence repeats
ATP	Adenosine triphosphate
NADH	Nicotinamide adenine dinucleotide
