# Supplementary material for: Comparative and Phylogenetic Analyses of Ginger (Zingiber officinale) in the Family Zingiberaceae Based on the Complete Chloroplast Genome
Source: Plants (Basel). 2019 Aug 12;8(8):283. doi: 10.3390/plants8080283 (PMC6724139; doi:10.3390/plants8080283)
Supplement: Supplementary file 1 [file plants-08-00283-s001.pdf]

Supplementary Material

Table S1. Gene with introns in *Z. officinale* chloroplast genome.

Gene	Location	Exon I (bp)	Intron I (bp)	Exon II (bp)	Intron II (bp)	Exon III (bp)
atpF	LSC	144	809	411		
clpP	LSC	69	826	296	629	253
ndhA	SSC	553	1061	539		
ndhB	IR	777	700	756		
petB	LSC	6	784	642		
petD	LSC	8	746	475		
rpl16	LSC	9	1051	402		
rpl2	IR	397	665	431		
rpoC1	LSC	428	732	1627		
rps7	IR	231	540	27		
rps12*	LSC	114	-	231	541	27
rps16	LSC	40	732	212		
trnA-UGC	IR	38	801	35		
trnG-UCC	LSC	23	682	41		
trnI-GAU	IR	42	935	35		
trnK-UUU	LSC	35	2591	37		
trnL-UAA	LSC	35	519	50		
trnV-UAC	LSC	38	606	37		
trnV-GAC	IR	27	540	231		
ycf3	LSC	127	715	230	806	153

Table S2. Codon usage of *Z. officinale* chloroplast genome.

Amino acid	Codon	No.	RSCU	tRNA	Amino acid	Codon	No.	RSCU	tRNA
Phe	UUU	952	1.28		Tyr	UAU	792	1.57	
Phe	UUC	531	0.72	trnF-GAA	Tyr	UAC	214	0.43	trnY-GUA
Leu	UUA	865	1.9	trnL-UAA	Stop	UAA	49	1.71	
Leu	UUG	575	1.26	trnL-CAA	Stop	UAG	23	0.8	
Leu	CUU	583	1.28		His	CAU	497	1.57	
Leu	CUC	178	0.39		His	CAC	138	0.43	trnH-GUG
Leu	CUA	374	0.82	trnL-UAG	Gln	CAA	700	1.53	trnQ-UUG
Leu	CUG	157	0.34		Gln	CAG	215	0.47	
Ile	AUU	1155	1.49		Asn	AAU	984	1.55	
Ile	AUC	420	0.54	trnI-GAU	Asn	AAC	289	0.45	trnN-GUU
Ile	AUA	756	0.97	trnI-CAU	Lys	AAA	1070	1.47	trnK-UUU
Met	AUG	616	1	trn(f)M-CAU	Lys	AAG	381	0.53	
Val	GUU	509	1.44		Asp	GAU	881	1.66	
Val	GUC	164	0.46	trnV-GAC	Asp	GAC	181	0.34	trnD-GUC
Val	GUA	547	1.54	trnV-UAC	Glu	GAA	1063	1.5	trnE-UUC
Val	GUG	198	0.56		Glu	GAG	355	0.5	
Ser	UCU	600	1.73		Cys	UGU	237	1.56	
Ser	UCC	338	0.97	trnS-GGA	Cys	UGC	66	0.44	trnC-GCA
Ser	UCA	419	1.21	trnS-UGA	Stop	UGA	14	0.49	
Ser	UCG	187	0.54		Trp	UGG	456	1	trnW-CCA
Pro	CCU	444	1.65		Arg	CGU	365	1.36	trnR-ACG
Pro	CCC	200	0.74		Arg	CGC	88	0.33	
Pro	CCA	307	1.14	trnP-UGG	Arg	CGA	342	1.27	
Pro	CCG	123	0.46		Arg	CGG	110	0.41	
Thr	ACU	531	1.58		Arg	AGA	438	1.26	trnR-UCU
Thr	ACC	232	0.69	trnT-GGU	Arg	AGG	103	0.3	
Thr	ACA	421	1.25	trnT-UGU	Ser	AGU	533	1.99	
Thr	ACG	158	0.47		Ser	AGC	173	0.64	trnS-GCU
Ala	GCU	615	1.81		Gly	GGU	602	1.39	
Ala	GCC	208	0.61		Gly	GGC	139	0.32	trnG-GCC
Ala	GCA	423	1.25	trnA-UGC	Gly	GGA	717	1.65	trnG-UCC
Ala	GCG	111	0.33		Gly	GGG	277	0.64	

RSCU: Relative Synonymous Codon Usage.

Table S3. Simple sequence repeats (SSRs) in *Z. officinale* chloroplast genome.

specie	SSR nr.	SSR type	SSR	size	start	end
A. villosum	1	p2	(AT)5	10	2633	2642
	2	p3	(AAT)4	12	3437	3448
	3	p1	(T)11	11	5487	5497
	4	p1	(T)10	10	6535	6544
	5	p1	(A)11	11	7091	7101
	6	p1	(T)10	10	8391	8400
	7	p4	(TTAT)3	12	12508	12519
	8	p1	(T)10	10	12692	12701
	9	p3	(AAT)4	12	12745	12756
	10	p1	(T)12	12	13455	13466
	11	p4	(TTTA)3	12	13475	13486
	12	p2	(AT)7	14	14568	14581
	13	p4	(TTAT)3	12	14695	14706
	14	p4	(TTAT)3	12	14715	14726
	15	p2	(AT)6	12	14853	14864
	16	p2	(AT)9	18	14936	14953
	17	p1	(A)10	10	15479	15488
	18	p1	(T)10	10	17240	17249
	19	p1	(T)12	12	19477	19488
	20	p1	(A)10	10	19620	19629
	21	p2	(AT)5	10	20848	20857
	22	p1	(A)10	10	28502	28511
	23	p1	(A)13	13	30841	30853
	24	p4	(ATTT)3	12	33169	33180
	25	p2	(TA)5	10	33222	33231
	26	p3	(TCT)4	12	34486	34497
	27	p1	(T)10	10	37629	37638
	28	p2	(AT)5	10	37960	37969
	29	p2	(TA)6	12	38117	38128
	30	p1	(T)11	11	39319	39329
	31	p2	(TA)7	14	48482	48495
	32	p2	(AT)7	14	49702	49715
	33	p4	(CAAA)3	12	49807	49818
	34	p2	(AT)5	10	50974	50983
	35	p2	(AT)5	10	53497	53506
	36	p4	(ATTT)3	12	55438	55449
	37	p1	(A)11	11	60047	60057
	38	p2	(AT)5	10	62346	62355
	39	p2	(AT)6	12	62530	62541
	40	p4	(CTAA)3	12	63880	63891

41	p4	(ATAA)3	12	65034	65045
42	p4	(AGAA)3	12	66556	66567
43	p2	(AT)6	12	68393	68404
44	p4	(GAAA)3	12	68474	68485
45	p4	(GAAA)3	12	68487	68498
46	p4	(TATT)3	12	69405	69416
47	p1	(T)11	11	69474	69484
48	p1	(A)10	10	71639	71648
49	p5	(TTCTA)3	15	73689	73703
50	p1	(T)10	10	73874	73883
51	p5	(AATAA)3	15	74045	74059
52	p4	(ATAA)3	12	74334	74345
53	p1	(T)10	10	85398	85407
54	p4	(TTCT)3	12	85884	85895
55	p2	(GA)5	10	93505	93514
56	p1	(T)13	13	103019	103031
57	p1	(T)10	10	115466	115475
58	p4	(ATTT)3	12	116237	116248
59	p4	(GAAT)3	12	117021	117032
60	p2	(AT)5	10	119915	119924
61	p2	(TA)5	10	120624	120633
62	p3	(TAA)4	12	120996	121007
63	p4	(AATA)3	12	122777	122788
64	p2	(AT)5	10	124856	124865
65	p2	(AT)5	10	124868	124877
66	p2	(TA)6	12	124995	125006
67	p1	(A)10	10	126206	126215
68	p4	(AAAT)3	12	126408	126419
69	p3	(TTA)4	12	130904	130915
70	p1	(T)12	12	131697	131708
71	p1	(T)12	12	132233	132244
72	p1	(T)12	12	132366	132377
73	p2	(AT)5	10	132801	132810
74	p4	(CATT)3	12	133075	133086
75	p4	(AAAT)3	12	133860	133871
76	p1	(A)10	10	134633	134642
77	p1	(A)13	13	147077	147089
78	p2	(TC)5	10	156594	156603

Table S4. Primer sequences at the boundaries between single copy and IR regions of Z. *officinale*.

Regions	Forward/Reverse	Primer sequence (5' to 3')
LSC-IRa	F	TGACAAGTGGGACTTTTTATACCAT
	R	CTTGGTCTCGGGCATCTACT
IRa-SSC	F	ATCGCGGTACCCATCCTTTT
	R	CCCAATGTGAGTTATTCCGTGG
SSC-IRb	F	TCAAGTTTGGCACCGATATGT
	R	ATATCGCGGTACCCATCCTT
IRb-LSC	F	TAGTAACTTGGTCTCGGGCA
	R	CAGATTCGGCCAAGAGGAAGA

Table S5. GenBank accession numbers of 19 complete chloroplast genome sequences used for ML phylogenetic analyses.

GenBank acc.	Species	GenBank acc.	Species
NC_020363	*Zingiber spectabile*	NC_022927	*Ravenala madagascariensis*
NC_040852	*Kaempferia elegans*	NC_028439	*Musa balbisiana*
NC_040851	*Kaempferia galanga*	NC_035723	*Musa itinerans*
NC_028729	*Curcuma flaviflora*	NC_022926	*Musa textilis*
NC_022928	*Curcuma roscoeana*	HF677508.1	*Musa acuminata subsp. malaccensis*
MK919702	*Curcuma longa*	NC_035637	*Musella lasiocarpa*
NC_036935	*Amomum krervanh*	NC_020362	*Heliconia collinsiana*
NC_036992	*Amomum compactum*	MG267380	*Dioscorea polystachya*
NC_035895	*Alpinia oxyphylla*	NC_037717	*Dioscorea collettii*
